# Increase in serum albumin concentration is associated with prediabetes development and progression to overt diabetes independently of metabolic syndrome

**DOI:** 10.1371/journal.pone.0176209

**Published:** 2017-04-21

**Authors:** Ji Eun Jun, Seung-Eun Lee, You-Bin Lee, Jae Hwan Jee, Ji Cheol Bae, Sang-Man Jin, Kyu Yeon Hur, Moon-Kyu Lee, Jae Hyeon Kim

**Affiliations:** 1 Division of Endocrinology and Metabolism, Department of Medicine, Samsung Medical Center, Sungkyunkwan University School of Medicine, Gangnam-gu, Seoul, Republic of Korea; 2 Department of Health Promotion Center, Samsung Medical Center, Sungkyunkwan University School of Medicine, Seoul, Republic of Korea; 3 Division of Endocrinology and Metabolism, Department of Medicine, Samsung Changwon Hospital, Sungkyunkwan University School of Medicine, Changwon, Republic of Korea; 4 Department of Clinical Research Design & Evaluation, SAIHST, Sungkyunkwan University School of Medicine, Seoul, Republic of Korea; Mathematical Institute, HUNGARY

## Abstract

**Aim:**

Serum albumin concentration is associated with both type 2 diabetes and metabolic syndrome (MetS). We sought to investigate whether baseline serum albumin and change in serum albumin could be independent risk factors for prediabetes in subjects without MetS. We further examined the effect of serum albumin on progression to overt diabetes in subjects who developed prediabetes.

**Methods:**

Among 10,792 participants without diabetes and MetS who consecutively underwent yearly health check-ups over six years, 9,807 subjects without incident MetS were enrolled in this longitudinal retrospective study. The risk of developing prediabetes (impared fasting glucose or hemoglobin A1c) was analyzed according to baseline and percent change in serum albumin concentration using Cox regression analysis. Serial changes in serum albumin concentration were measured from baseline to one year before prediabetes diagnosis, and then from the time of prediabetes diagnosis to progression to overt diabetes or final follow-up.

**Results:**

A total of 4,398 incident cases of prediabetes developed during 35,807 person-years (median 3.8 years). The hazard ratio for incident prediabetes decreased as percent change in serum albumin concentration (quartiles and per 1%) increased in a crude and fully adjusted model. However, baseline serum albumin concentration itself was not associated with prediabetic risk. Serum albumin levels kept increasing until the end of follow-up in prediabetic subjects who returned to normal glycemic status, whereas these measures did not change in prediabetic subjects who developed type 2 diabetes. Serum albumin concentration measured at the end of follow-up was the highest in the regression group, compared to the stationary (p = 0.014) or progression groups (p = 0.009).

**Conclusions:**

Increase in serum albumin concentration might protect against early glycemic deterioration and progression to type 2 diabetes even in subjects without MetS.

## Introduction

Serum albumin is the most abundant plasma protein and it provides oncotic pressure, transports bilirubin and other hormones [1], and serves as an extracellular antioxidant agent [2–4]. Based on the potential antioxidant properties of serum albumin, emerging evidence indicates that serum albumin concentration might be associated with metabolic disorders such as type 2 diabetes and metabolic syndrome (MetS). Low serum albumin concentration enhanced the risk for incident type 2 diabetes [5–7]; however, inverse correlations have been reported as well [8]. Serum albumin concentration was positively associated with prevalence of MetS [9,10], whereas increase in serum albumin over time might protect against MetS development [11].

Previous studies that investigated associations between serum albumin and diabetes included subjects with MetS at baseline or incident MetS during the follow-up period, indicating that some portion of the included participants was already susceptible to developing type 2 diabetes, or that independent association between serum albumin and diabetes was partially interrupted by incident MetS before diabetes development. In fact, one longitudinal study showed paradoxical contributions to incident MetS by baseline serum albumin concentration and changes in serum albumin [11]. Additionally, declines in insulin sensitivity had a greater effect on diabetes development than did insulin resistance, especially in Asian populations [12,13]. However, the association between serum albumin and glycemic alteration in subjects without evident insulin resistance has rarely been examined.

Herein, we sought to investigate whether baseline and change in serum albumin levels during an observation period could be independent risk factors for prediabetes especially impaired fasting glucose (IFG) or hemoglobin A1c (IA1c) in subjects without baseline and new MetS. We further examined the effect of serum albumin on the risk for progression to overt diabetes in prediabetic subjects.

## Materials and methods

### 1. Study population

In all, 24,185 adults underwent voluntary comprehensive health check-ups between January 2006 and December 2012 at Samsung Medical Center. The health check-up programs included anthropometric data, laboratory data, and questionnaires about patient medical history and lifestyle. From the baseline data, which were collected when the participants first visited the hospital, 13,393 individuals were excluded for the following reasons (S1 Fig): 1) baseline type 2 diabetes (*n* = 1,321), prediabetes especially IFG or IA1c (*n* = 4,736) or MetS (*n* = 962); 2) history of cardiovascular disease including myocardial infarction or stroke (*n* = 692); 3) new onset type 2 diabetes (*n* = 28) and prediabetes (*n* = 2,053) within one year of follow-up; 4) diagnosis of type 2 diabetes prior to receiving an prediabetes diagnosis (*n* = 23); 5) < 20 years old (*n* = 4); 6) abnormal liver function (*n* = 338), which was defined as either elevated levels of total bilirubin, aspartate aminotransferase (AST) or alanine aminotransferase (ALT) that was more than twice the upper normal limit, or being positive for the hepatitis B surface antigen (*n* = 955) or hepatitis C antibody (*n* = 191); 7) estimated glomerular filtration rate (GFR) < 60 ml/min/1.73m^2^, calculated using the Chronic Kidney Disease Epidemiology Collaboration (CKD-EPI) formula (*n* = 180); 8) missing clinical variables (*n* = 1,287); and 9) development of MetS prior to prediabetes onset (*n* = 985). No subjects showed evidence of cancer or chronic infection in their electronic records. Thus, the final number of participants in our study was 9,807 (4,628 men and 5,179 women).

This study was approved by the Institutional Review Board of Samsung Seoul Hospital and carried out in accordance with the recommendations of the Declaration of Helsinki.

### 2. Definitions

Prediabetes was defined as fasting plasma glucose (FPG) 100–125 mg/dl (IFG) or HbA1c 5.7–6.4% (IA1c). Diabetes was defined as FPG ≥ 126 mg/dl or HbA1C ≥ 6.5%. Subjects who were using insulin or oral antidiabetic drugs based on a self-report questionnaire given at each visit were also considered to have diabetes. The definition for MetS was established according to harmonized criteria from related federations [14]. Three or more of the following criteria constituted a diagnosis of MetS: 1) abdominal obesity (waist circumference [WC] ≥ 90 cm in men, and ≥ 80 cm in women); 2) systolic blood pressure (SBP) ≥ 130 mmHg, diastolic blood pressure (DBP) ≥ 85 mmHg, or currently taking anti-hypertensive medications; 3) triglycerides (TG) ≥ 150 mg/dl; 4) high density lipoprotein cholesterol (HDL-C) < 40 mg/dl in men or < 50 mg/dl in women; 5) FPG ≥ 100 mg/dl or use of anti-diabetic drugs. In contrast, normal weight was defined as BMI < 23 kg/m^2^, and the normal range for insulin resistance was < 2.5 [15].

Percent change in albumin and body mass index (BMI) was obtained with the following formula: (final value—baseline value)/baseline value x 100 [11]. The final value was measured at the end of follow-up in subjects without incident prediabetes or one year before prediabetes diagnosis. Subjects with incident prediabetes was further classified to three groups according to their final metabolic status. The regression group included subjects whose fasting glucose and HbA1c returned to normal one or two years after prediabetes diagnosis, and then remained normal until the end of follow-up. The stationary group included subjects who remained prediabetic until the end of follow-up, and the progression group included subjects who developed type 2 diabetes.

### 3. Clinical and biochemical measurements

Demographic characteristics were assessed using a structured questionnaire at the first medical check-up. Blood pressure was measured at each visit by trained nurses using a mercury sphygmomanometer on the right arm while the participants sat in a comfortable position after at least 5 minutes of rest. BMI was calculated by dividing body weight by the height squared (kg/m^2^).

Venous blood samples were collected from the antecubital vein after an overnight fast, and all of the laboratory tests were carried out in the same central certified laboratory at Samsung Medical Center. Serum albumin levels were measured using the bromocresol green dye-binding method on a Roche Modular DP analyzer (Roche Diagnostics, Basel, Switzerland). HbA1c was measured using high performance liquid chromatography on an HLC-723G8 automated glycohemoglobin analyzer (TOSOH, Yokkaichi, Japan) that had been standardized to the reference method, which aligned with standards set by the Diabetes Control and Complication Trial and the National Glycohemoglobin Standardization Program. Plasma glucose level was determined using the hexokinase method with a GLU kit (Roche Diagnostics) on a Roche Modular DP analyzer (Roche Diagnostics). Participants’ insulin levels were obtained through immunoradiometric assays (Biosource, Nivelles, Belgium), and insulin resistance, for which we used Homeostatic Model Assessment Index for Insulin Resistance (HOMA-IR) system, was calculated using the following formula: fasting insulin (μIU/ml) × fasting glucose (mmol/l) / 22.5 [16].

### 4. Statistical analyses

Normally distributed data were expressed as mean ± standard deviation (SD), while unevenly distributed data were presented as medians (interquartile range: 25^th^ to 75^th^ percentile) for continuous variables and percentages for categorical variables. One-way analysis of variance (ANOVA) was used to compare continuous variables and the Chi-square test was used to analyze differences among quartiles of baseline and percent change in albumin level and other categorical variables.

We used multivariate Cox regression analysis to assess the hazard ratio (HR) of prediabetes according to quartiles of serum albumin level and also for continuous variables per 1 mg/dl or 1 percent. Each Cox regression model was adjusted for potential metabolic risk factors: Model 1 was adjusted for age, gender and BMI; Model 2 was additionally adjusted for fastng glucose and HbA1c; Model 3 was further adjusted for ALT, triglyceride (TG), low-density lipoprotein cholesterol (LDL-C), high-density lipoprotein cholesterol (HDL-C), hypertension, and smoking status. Since data for C-reactive protein (CRP) were available for only 9,789 subjects, log CRP was added as a covariate in model 4. Model 5 was further adjusted for HOMA-IR which were available in 6,222 subjects. All covariates in the multivariate logistic models had a variance inflation factor < 2.0, which was considered adequate to avoid relevant multicollinearity. Linear regression analyses were also perfomed to check possible association between serum albumin and various metabolic parameters measured at baseline and prediabetes diagnosis. Receiver operating curve (ROC) analyses were used to compare the predictive power of serum albumin and other variables for diagnosing prediabetes or type 2 diabetes.

All statistical tests were two-tailed, and analyses were executed using SPSS for Windows version 23.0 (Chicago, IL, USA). A *p*-value < 0.05 was considered statistically significant.

## Results

### 1. Baseline characteristics of total subjects according to baseline serum albumin and percent change in serum albumin level

The clinical characteristics of the study population according to baseline serum albumin quartiles are summarized in S1 Table. During 35,807 person-years of follow-up (median 3.8 years), 4,398 new cases (44.8%) of MetS were observed. However, the incidence of MetS was not statistically different across baseline albumin quartiles. Subjects were younger and had less favorable metabolic parameters (i.e., higher abdominal obesity, increased level of TC, TG, LDL-C, fasting glucose, HOMA-IR, and CRP levels), as baseline albumin quartiles increased. The proportion of current smokers increased and total body fat percent decreased across baseline albumin quartiles, as the proportion of men increased.

Clinical characteristics according to percent change in serum albumin quartiles are presented in Table 1. The incidence of MetS significantly decreased across percent change in serum albumin quartiles (p < 0.001). The proportion of men and current smokers, waist circumference, BMI, fasting glucose, HbA1c, HOMA-IR, TC, TG, LDL-C, and HDL-C decreased across percent change in serum albumin quartiles, while body fat percent increased. Baseline serum albumin level also decreased as the percent change in serum albumin quartiles increased.

**Table 1:** Clinical characteristics according to percent changes in serum albumin.

Variables	Quartile 1 < -2.08%	Quartile 2 -2.08–2.43%	Quartile 3 2.44–7.31%	Quartile 4 > 7.31%	p value
Number of subject	2436	2368	2476	2527	
Age (year)	49.88 ± 7.84	48.88 ± 7.93	49.05 ± 7.82	48.62 ± 7.37	< 0.001
Male (n, %)	1325 (54.4)	1202 (50.8)	1132 (45.7)	969 (38.3)	< 0.001
Waist circumference (cm)	80.39 ± 8.78	79.78 ± 8.59	79.12 ± 8.50	77.94 ± 8.54	< 0.001
BMI (kg/m^2^)	22.91 ± 2.48	22.74 ± 2.46	22.68 ± 2.46	22.44 ± 2.41	< 0.001
Body fat (%)	23.63 ± 5.93	23.68 ± 6.13	24.01 ± 6.12	24.19 ± 5.98	0.002
Hypertension (n, %)	193 (7.9)	185 (7.8)	177 (7.1)	168 (6.6)	0.056
Current smoker (n, %)	359 (14.7)	315 (13.3)	348 (14.1)	296 (11.7)	< 0.001
Fasting glucose (mg/dl)	85.44 ± 6.93	84.79 ± 6.67	84.16 ± 6.92	83.35 ± 6.78	< 0.001
HbA1c (%)	5.20 ± 0.26	5.16 ± 0.27	5.14 ± 0.26	5.12 ± 0.27	< 0.001
HOMA-IR (n = 6,222)	1.76 ± 0.74	1.72 ± 0.69	1.71 ± 0.68	1.65 ± 0.68	< 0.001
Basseline albumin (mg/dl)	4.43 ± 0.22	4.36 ±0.19	4.24 ± 0.20	4.11 ± 0.20	< 0.001
ALT (U/l)	20.43 ± 9.75	19.81 ± 9.64	19.09 ± 9.56	18.03 ± 9.15	< 0.001
Total cholesterol (mg/dl)	191.95 ± 30.43	189.70 ± 30.63	186.73 ± 29.90	182.16 ± 30.87	< 0.001
LDL-C (mg/dl)	123.58 ± 27.84	121.27 ± 27.21	118.12 ± 26.61	114.49 ± 27.10	< 0.001
HDL-C (mg/dl)	61.61 ± 14.09	61.53 ± 13.76	61.22 ± 13.72	60.19 ± 13.57	0.001
TG (mg/dl)	106.68 ± 58.38	103.42 ± 51.27	100.85 ± 49.24	97.93 ± 48.59	< 0.001
eGFR (ml/min/1.73 m^2^)	88.72 ± 11.77	90.33 ± 12.56	90.10 ± 12.14	90.77 ± 12.48	< 0.001
CRP (mg/dl) (n = 9,789)	0.05 (0.03–0.09)	0.05 (0.03–0.09)	0.05 (0.03–0.09)	0.05 (0.03–0.09)	0.792
Incidence of prediabetes, n (%)	1567 (64.3)	1118 (47.2)	922 (37.2)	791 (31.3)	< 0.001

Data are presented as mean ± SD, median (25^th^ to 75^th^ percentile) or percentages. p value was calculated from one way analysis of variance (ANOVA) or the Kruskal-Wallis test for continuous variables or the Chi-square test for categorical variables. Abbreviations: BMI, body mass index; HOMA-IR, homeostatic model assessment-insulin resistance; ALT, alanine aminotransferase; LDL-C, low-density lipoprotein cholesterol, HDL-C;high-density lipoprotein cholesterol; TG, triglyceride; eGFR, estimated glomerular filtration rate; CRP, C-reactive protein.

### 2. Comparison of baseline and final levels of serum albumin between subjects with and without incident prediabetes

**Fig 1:**
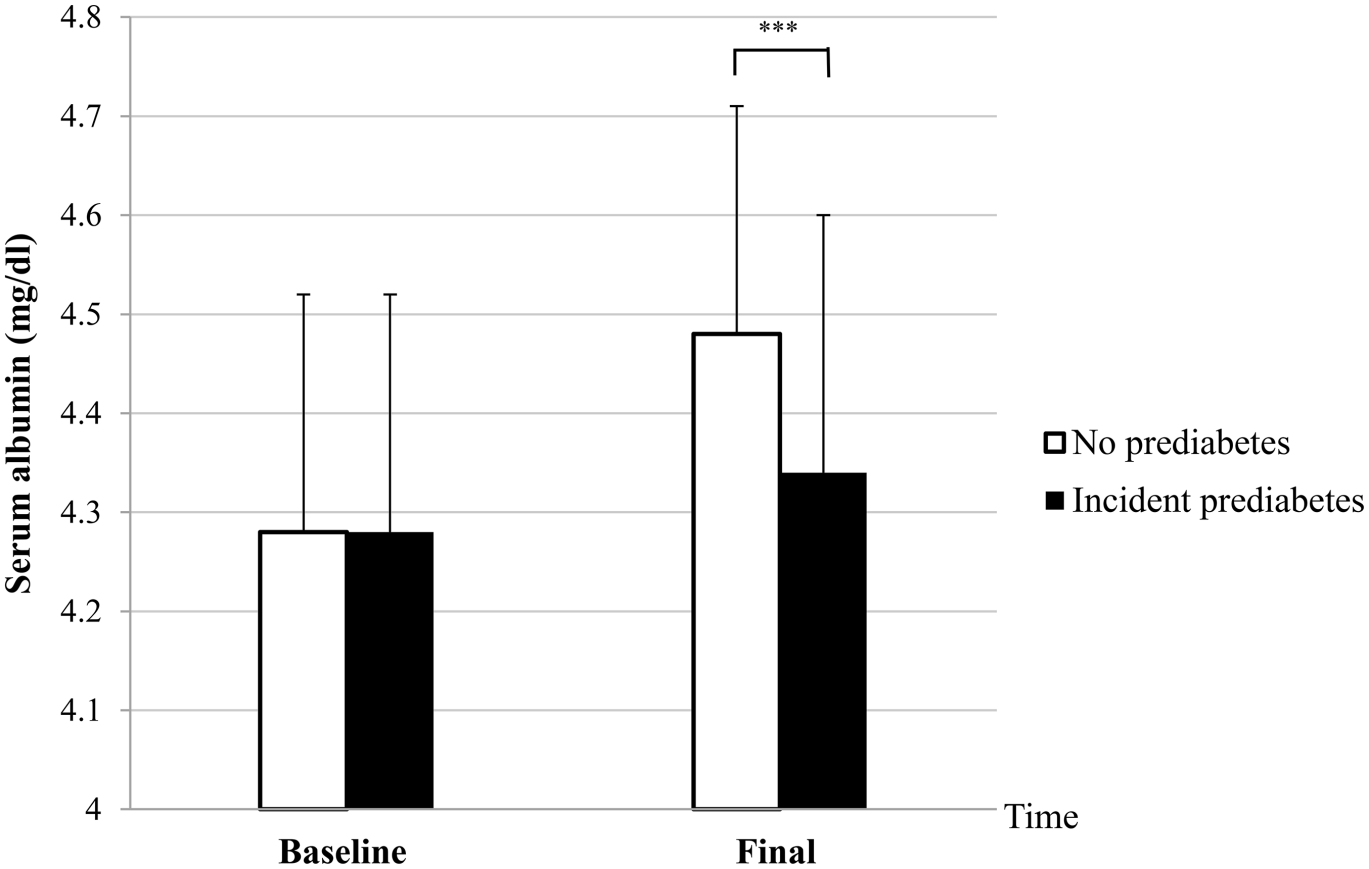
Comparison of baseline and final serum albumin levels between subjects with and without incident prediabetes. *p < 0.05, **p < 0.01, ***p < 0.001 by t-test.

There was no difference in baseline serum albumin levels between the two groups. However, final serum albumin levels increased significantly in subjects who did not develop prediabetes (4.48 ± 0.24 vs 4.34 ± 0.26 mg/dl, p < 0.001), compared to subjects who developed prediabetes as shown in Fig 1. Percent change in serum albumin was also apparently higher in subjects without incident prediabetes (4.77 ± 6.20 vs 1.42 ± 6.31%, p < 0.001).

### 3. Association between serum albumin and glycemic parameters measured at baseline versus at prediabetes diagnosis

Linear regression analysis showed a positive linear relationship between baseline serum albumin level and baseline fasting glucose level (standardized β = 0.125, p < 0.001), while there was no correlation between baseline serum albumin level and baseline HbA1c level. Baseline serum albumin was positively associated with baseline insulin (standardized β = 0.058, p < 0.001). However, inverse associations were found between serum albumin measured at prediabetes diagnosis and fasting glucose (standardized β = -0.043, p < 0.001) or HbA1c measured at prediabetes diagnosis (standardized β = -0.213, p < 0.001). In addition, serum albumin change showed a strong inverse correlation between fasting glucose (standardized β = -0.164, p < 0.001) or HbA1c (standardized β = -0.116, p < 0.001) measured at prediabetes diagnosis, whereas baseline serum albumin was not associated with fasting glucose or HbA1c measured at prediabetes diagnosis.

### 4. Association between baseline serum albumin or percent change in serum albumin and prediabetes development

The overall incidence of prediabetes in this cohort was 44.8% over a median 3.84 years. The incidence of prediabetes increased across percent change in serum albumin quartiles (p for trend < 0.001) even though it was not different across baseline albumin quartiles in Table 2. In all crude and multivariate Cox regression models (models 1–5), baseline serum albumin as quartiles and continuous values was not associated with risk for developing prediabetes (Table 2). However, a higher percent change in serum albumin was significantly associated with decreasing risk of incident prediabetes in crude and multivariate Cox regression models (models 1–5) as both quartiles and continuous values (Table 2). Percent change in serum albumin levels remained an independent protective factor for incident prediabetes (models 1–5) in subjects with normal BMI and waist circumference and who did not have insulin resistance (Table 3). When total subjects were divided two subgroups according to the age of ≥ 60 which had highest prevalence of diabetes, increase in serum albumin concentration was still significantly associated with lower risk of prediabetes development (S2 Table).

**Table 2:** Hazard ratios and 95% confidence intervals for prediabetes development according to baseline and percent changes in serum albumin.

Baseline levels of serum albumin	Quartile 1 (< 4.1 mg/dl)	Quartile 2 (4.1–4.2 mg/dl)	Quartile 3 (4.3–4.4 mg/dl)	Quartile 4 (> 4.4 mg/dl)	p for trend	Cont.variable (per 1 mg/dl)	p value
Incident prediabetes, n (%)	678 (43.5)	1225 (43.4)	1522 (47.7)	973 (43.5)	0.283		
Crude	1	1.00 (0.91–1.10)	1.02 (0.93–1.13)	1.13 (1.04–1.24)	0.137	1.067 (0.941–1.209)	0.313
Multivariate 1	1	1.02 (0.93–1.12)	1.07 (0.97–1.19)	1.15 (1.05–1.26)	0.025	1.137(0.998–1.296)	0.054
Multivariate 2	1	1.01 (0.92–1.11)	0.99 (0.89–1.09)	1.08 (0.99–1.19)	0.814	0.971 (0.852–1.108)	0.665
Multivariate 3	1	1.00 (0.91–1.10)	0.94 (0.85–1.05)	1.04 (0.95–1.14)	0.439	0.905 (0.790–1.037)	0.152
Multivariate 4	1	1.00 (0.91–1.10)	1.00 (0.95–1.14)	0.95 (0.85–1.05)	0.461	0.911 (0.794–1.044)	0.178
Multivariate 5	1	1.00 (0.89–1.12)	1.02 (0.91–1.15)	0.98 (0.86–1.11)	0.834	0.934 (0.786–1.110)	0.440
Serum albumin change (%)	Quartile 1 (< - 2.08%)	Quartile 2 (-2.08–2.43%)	Quartile 3 (2.44–7.31%)	Quartile 4 (> 7.31%)	p for trend	Cont.variable (per 1%)	p value
Incident prediabetes, n (%)	1567 (64.3)	1118 (47.2)	922 (37.2)	791 (31.3)	< 0.001		
Crude	1	0.60 (0.56–0.64)	0.43 (0.40–0.47)	0.33 (0.31–0.36)	< 0.001	0.932 (0.927–0.936)	< 0.001
Multivariate 1	1	0.61 (0.57–0.65)	0.44 (0.41–0.47)	0.35 (0.32–0.38)	< 0.001	0.934 (0.929–0.938)	< 0.001
Multivariate 2	1	0.66 (0.62–0.71)	0.48 (0.45–0.52)	0.40 (0.37–0.43)	< 0.001	0.942 (0.937–0.946)	< 0.001
Multivariate 3	1	0.66 (0.62–0.71)	0.48 (0.45–0.52)	0.40 (0.37–0.43)	< 0.001	0.942 (0.937–0.946)	< 0.001
Multivariate 4	1	0.67 (0.62–0.73)	0.48 (0.44–0.52)	0.40 (0.37–0.44)	< 0.001	0.942 (0.938–0.947)	< 0.001
Multivariate 5	1	0.67 (0.61–0.74)	0.46 (0.42–0.51)	0.38 (0.34–0.42)	< 0.001	0.939 (0.934–0.945)	< 0.001

Data are expressed as hazard ratio (95% confidence interval). Model 1: adjusted for age, gender, and BMI. Model 2: adjusted for Model 1 plus fasting plasma glucose and HbA1c. Model 3: adjusted for Model 2 plus ALT, TG, LDL-C, HDL-C, eGFR, hypertension and smoking status. Model 4: adjusted for Model 3 plus logCRP (n = 9,789). Model 5: adjusted for Model 4 plus HOMA-IR (n = 6,222).

**Table 3:** Hazard ratios and 95% confidence intervals for prediabetes development according to percent changes in serum albumin level in subjects without obesity or insulin resistance.

Serum albumin change (per 1%)						
	^a^Abdominal obesity (-) (n = 6,725)	p value	BMI < 23 (n = 5,431)	p value	HOMA-IR < 2.5 (n = 5,504)	p value
Crude	0.931 (0.926–0.937)	< 0.001	0.933 (0.926–0.939)	< 0.001	0.930 (0.924–0.936)	< 0.001
Multivariate 1	0.932 (0.927–0.938)	< 0.001	0.934 (0.928–0.941)	< 0.001	0.932 (0.926–0.938)	< 0.001
Multivariate 2	0.939 (0.934–0.945)	< 0.001	0.942 (0.936–0.949)	< 0.001	0.939 (0.933–0.945)	< 0.001
Multivariate 3	0.939 (0.933–0.945)	< 0.001	0.942 (0.936–0.949)	< 0.001	0.939 (0.933–0.945)	< 0.001
Multivariate 4	0.938 (0.933–0.944)	< 0.001	0.942 (0.936–0.949)	< 0.001	0.939 (0.932–0.945)	< 0.001
Multivariate 5	0.935 (0.928–0.942)	< 0.001	0.940 (0.932–0.948)	< 0.001	0.939 (0.933–0.945)	< 0.001

^a^Abdominal obesity was defined as waist circumference ≥ 90 cm for men and ≥ 80 cm for women.

Data are expressed as hazard ratio (95% confidence interval). Model 1: adjusted for age, gender, and BMI. Model 2: adjusted for Model 1 plus fasting plasma glucose and HbA1c. Model 3: adjusted for Model 2 plus ALT, TG, LDL-C, HDL-C, eGFR, hypertension and smoking status. Model 4: adjusted for Model 3 plus logCRP (n = 9,789). Model 5: adjusted for Model 4 plus HOMA-IR (n = 6,222).

### 5. Percent change in BMI and percent change in serum albumin

All subjects were classified into three groups according to tertiles of BMI percent change over the observation period: decreasing BMI group (-24.07 to -1.33%), unchanged group (-1.32 to 1.70%), and increasing BMI group (1.71 to 25.60%). Regardless of baseline BMI and BMI change, the subjects with incident prediabetes showed significantly lower percent change in serum albumin level, compared to normal subjects (Table 4). Change in serum albumin was higher in the BMI increasing group regardless of prediabetes development (Table 4). However, higher percent change in serum albumin levels was still significantly associatied with decreasing risk of new-onset prediabetes in the multivariate Cox regression models (models 1–5), even when baseline BMI and BMI change were concurrently adjusted (S3 Table).

**Table 4:** Relationship between serum albumin change and BMI change.

	BMI change group	Albumin change (%)		p value
		Incident prediabetes(-)	Incident prediabetes (+)	
Baseline BMI < 25 (n = 8,161)	decreasing BMI (*n* = 2,595)	4.52 ± 6.20	0.56 ± 6.30	< 0.001
	unchanged BMI (*n* = 2,711)	4.57 ± 6.12	1.74 ± 6.22	< 0.001
	increasing BMI (*n* = 2,855)	5.28 ± 6.36	2.29 ± 6.38	< 0.001
	p value	0.001	< 0.001	
Baseline BMI ≥ 25 (n = 1,646)	decreasing BMI (*n* = 672)	3.89 ± 5.53	0.28 ± 5.99	< 0.001
	unchanged BMI (*n* = 567)	4.46 ± 6.15	1.20 ± 6.22	< 0.001
	increasing BMI (*n* = 407)	5.04 ± 5.59	2.03 ± 6.37	0.001
	p value	0.140	0.006	

Decreasing BMI: First tertiles of BMI percent change in total subjects (−24.07 − −1.33%). Unchanged BMI: Second tertiles of BMI percent change in total subjects (-1.32–1.70%). Increasing BMI: Third tertiles of BMI percent change in total subjects (1.71–25.60%).

### 6. Serial changes in serum albumin from baseline to final follow-up in prediabetic subjects

Among 4,398 subjects who developed prediabetes, 3,851 subjects had a final serum albumin level that was measured at final follow-up or at the time of diabetes diagnosis. Of these, 1,316 subjects (34.2%) were included in the regression group which included subjects who returned to normal glucose tolerance after prediabetes diagnosis. Another 2,470 subjects (64.1%) were included in the stationary group, and 65 subjects (1.7%) were included in the progression group, which developed type 2 diabetes for a median 2.44 years after their prediabetes diagnosis.

**Fig 2:**
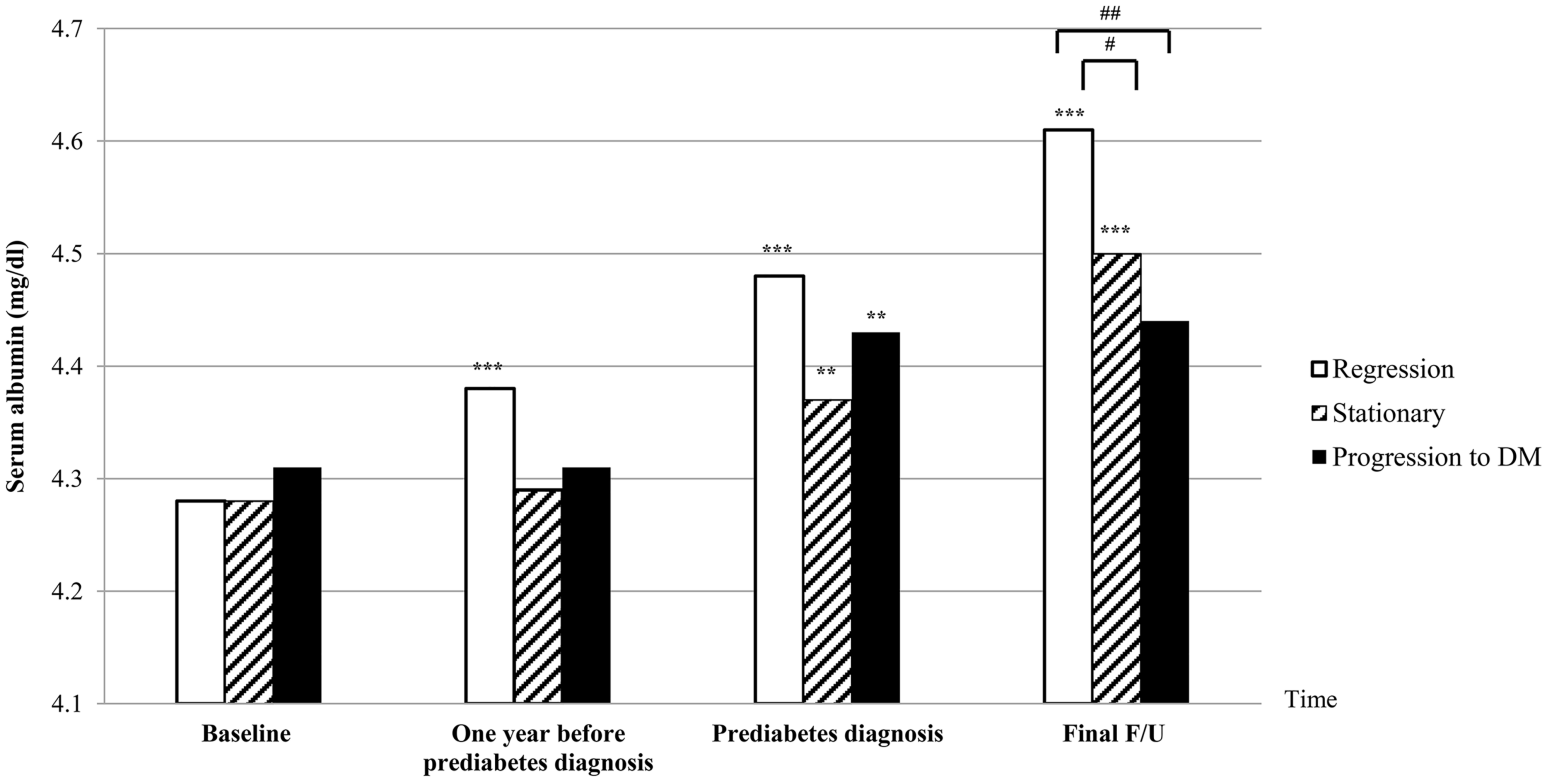
Changes in serum albumin levels from baseline to final follow-up in subjects with new-onset prediabetes. The regression group included subjects whose fasting glucose and HbA1c returned to normal one or two year after prediabetes diagnosis, and then remained normal until the end of follow-up. The stationary group included subjects who remained prediabetic until the end of follow-up, and the progression group included subjects who developed type 2 diabetes. *p < 0.05, **p < 0.01, ***p < 0.001 by paired t-test comparing two levels between the baseline and one year before prediabetes diagnosis, between the one year before and at the time of prediabetes diagnosis, and between prediabetes diagnosis and final follow-up. ^#^p < 0.05, ^##^p < 0.01, ^###^p < 0.001 in t-test. Duration from baseline to one-year before prediabetes diagnosis was 1.19 ± 0.97 year, duration from one-year before prediabetes diagnosis to diagnosis of IFG or IA1c was 1.04 ± 0.14 year, and duration from prediabetes diagnosis to final follow-up (diagnosis of diabetes or not) was 2.44 ± 1.09 year.

Although baseline serum albumin levels were not different, serum albumin levels measured before and after prediabetes diagnosis showed a distinct pattern among the three groups (Fig 2): Serum albumin levels kept increasing drastically until the end of follow-up in the regression group. Conversely, serum albumin levels did not increase until one year before prediabetes diagnosis in the stationary and progression groups, and then increased slightly at the time of prediabetes diagnosis. Then, serum albumin levels increased only in the stationary group at the end of follow-up while the progression group had no changes after prediabetes diagnosis. Serum albumin concentration measured at the end of follow-up was the highest in the regression group in Fig 2, compared to stationary (p = 0.014) and progression groups (p = 0.009).

### 7. Comparison of predictability for prediabetes and diabetes development between serum albumin change and other metabolic risk factors

Although the significance of percent change in serum albumin levels for predicting prediabetes did not surpass that of HbA1c which was the strongest glycemic parameter, change in serum albumin was a more significant risk factor for impaired glucose control compared to fasting glucose or BMI (S4 Table).

In Receiver operating characteristics (ROC) curve analysis showed the area under the ROC curve (AUC) for HbA1c was the largest among the variables, and it is significantly larger than those for serum albumin change in predicting both prediabetes (p < 0.001) and diabetes (p = 0.011) development (Table 5). The AUC of serum albumin change was significantly larger than those for age, BMI and fasting glucose for predicting prediabetes development (all p < 0.001). However, there was no difference between AUCs for serum albumin change and AUCs of age, BMI, and fasting glucose in predicting diabetes development.

**Table 5:** The comparison of area under an ROC curve (AUC) between traditional diabetic risk factors and serum albumin change.

	Prediabetes^#^			Diabetes^##^		
Variables	AUC	95% CI	p value	AUC	95% CI	p value
Age (year)	0.619	0.608–0.630	< 0.001	0.618	0.553–0.684	0.001
BMI (kg/m^2^)	0.609	0.598–0.630	< 0.001	0.679	0.620–0.738	< 0.001
Fasting glucose (mg/dL)	0.617	0.606–0.628	< 0.001	0.607	0.534–0.681	0.003
HbA1c (%)	0.710	0.700–0.720	< 0.001	0.728	0.674–0.782	< 0.001
Serum albumin change (%)	0.651	0.640–0.662	< 0.001	0.612	0.545–0.679	0.002

^**#**^HbA1c was 5.7–6.4% or fasting plasma glucose was 100–125 mg/dL.

^**##**^HbA1c was more than 6.5% or fasting plasma glucose was more than 126 mg/dL.

AUC, area under curve; CI, confidence intervak; BMI, body mass index.

## Discussion

Our data suggest that baseline serum albumin concentration was not associated with incidence of prediabetes in subjects without MetS. However, increase in serum albumin concentrations was protective against prediabetes development, regardless of baseline BMI, BMI change, or insulin resistant status. Increases in serum albumin protected against the transition from prediabetes to overt type 2 diabetes in subjects without MetS. Although serum albumin change’s predictive power for diagnosing prediabetes could not surpass that of HbA1c, it was stronger than those of fasting glucose, BMI, and age. Moreover, the direction of correlation between serum albumin and glycemic indices changed according to the observation points (baseline versus onset of metabolic disease). Threfore, our results emphasize the importance of serum albumin change for predicing the early glycemic abnormality instead of baseline serum albumin level itself.

Little is known about the relationship between serum albumin and prediabetes, rather than as a component of MetS, and there have been conflicting results regarding the association between serum albumin and diabetes. These discordant results might be partially due to differences in the proportion of enrolled subjects who already had baseline MetS and who developed MetS during the observation period, because serum albumin concentration has also been shown to be associated with MetS presence [9–11] and MetS is an independent risk factor for incident diabetes regardless of how it is defined [17]. However, we found that increases in serum albumin were still independently associated with a lower risk of incident IFG or IA1c after excluding subjects who had baseline MetS and new-onset MetS.

The protective effect of increasing serum albumin concentrations for impaired glucose control might be explained by albumin’s role as antioxidant. A high percentage of metal ions, which are potent generators of reactive oxygen species (ROS) can bind to albumin in order to not interact with hydrogen peroxide (H_2_O_2_); binding to H_2_O_2_ leads to the formation of deleterious hydroxyl radicals [3,18]. Additionally, serum albumin contains a single reduced cysteine residue (Cys34) that is directly able to scavenge hydroxyl radicals [19]. ROS impairs insulin-signaling pathways and induces cytotoxicity in pancreatic beta cells, which promotes development of diabetes.

In presence of oxidative stress, chronic low grade inflammation plays a crucial role in the pathogenesis of metabolic disorders [20]. C-reactive protein (CRP) is a prominent inflammation marker, and has been positively associated with various oxidative stress markers independently of traditional metabolic risk factors even in subjects without metabolic disease [21–23]. In fact, individual mean CRP levels during observation period were significantly higher in subjects with incident prediabetes than subjects without prediabetes (median 0.06, 25^th^–75^th^ percentile 0.04–0.11 vs median 0.05, 25^th^–75^th^ percentile 0.04–0.09, p < 0.001). Log-transformed mean CRP levels were inversely related with serum albumin change even after adjusting BMI change (Standardized β = -0.022, p = 0.029). Because CRP level is positively associated with various oxidative stress markers, this inverse correlation between serum albumin change and mean CRP level might support anti-oxidant role for serum albumin for preventing development of metabolic deteriorations against chronic inflammation, which increases oxidative stress.

Since aggravated oxidative stress and impaired redox status are already present in prediabetic patients [24,25], an increase in serum antioxidant level could be an early stage adaptation for glucose deterioration. In this study, BMI increased modestly as serum albumin quartiles increased, and the percent change in BMI was positively associated with percent change in serum albumin regardless of IFG or IA1c development, even though the degree of serum albumin change was significantly lower in subjects with incident IFG or IA1c. In accord with our study, high total bilirubin, considered a type of serum antioxidant, was associated with lower prediabetes prevalence [26]. Serum uric acid level rose in subjects with prediabetes, and then decreased in subjects with diabetes [27]. Therefore, serum antioxidant levels may be capable of instigating an early increase, prior to development of overt metabolic disease such as diabetes or cardiovascular disease, to compensate for the deleterious effects of oxidative stress. Consequently, their concentrations might decrease as antioxidants are rapidly consumed, since hyperglycemia itself can accelerate ROS production via glycation or lipoxidation processes, and auto-oxidation of glucose [28]. Previously reported associations between low serum albumin and higher risk of diabetes and cardiovascular disease are partially explained by this hypothesis. We also suppose that failure to increase serum albumin is associated with progression to metabolic unhealthy status, based on serial serum albumin decrease from baseline until diabetes development with inverse correlation between serum albumin change and diabetic parameters measured at the time of prediabetes diagnosis. However, this hypothesis should be confirmed by further studies.

Our study has several limitations. We did not use the two hour postload glucose test to diagnose diabetes and prediabetes, which may have led to underestimating the incidence of prediabetes during the observation period. Also, we were not able to analyze information about diet and exercise intensity and frequency. We may have introduced a selection bias due to our retrospective design, despite the fact that this longitudinal study contained a large sample. Our study subjects may not represent the general population as well. Finally, glycated albumin which is a strong predictor of diabetes was not measured concurrently.

## Conclusions

In conclusion, increase in serum albumin concentration was independently associated with lower risk of developing prediabetes especially IFG or IA1c even after eliminating the effect of metabolic syndrome. Serum albumin concentration continuously increased in subjects with prediabetes who returned to normal glycemic status, compared to those who developed overt type 2 diabetes. Therefore, the results of this longitudinal analysis indicate that increases in serum albumin concentration might protect against early glycemic deterioration and progression to overt diabetes.

## Supporting information

S1 Fig Selection of study subjects. (DOCX)

S1 Table Clinical characteristics according to baseline serum albumin levels. (DOCX)

S2 Table Hazard ratios of serum albumin change for prediabetes development according to age subgroup. (DOCX)

S3 Table Hazard ratios and 95% confidence intervals for prediabetes development according to percent change in serum albumin level after adjusting for both BMI and BMI change. (DOCX)

S4 Table Comparison between the effect of change in serum albumin and conventional metabolic risk factors on the risk for prediabetes. (DOCX)

## Abbreviations

ALT: alanine aminotransferase
BMI: body-mass index
CI: confidence interval
CRP: C-reactive protein
DBP: diastolic blood pressure
eGFR: estimated glomerular filtration rate
FPG: fasting plasma glucose
HbA1c: glycated hemoglobin
HDL-C: high-density lipoprotein cholesterol
HOMA-IR: homeostasis model assessment index for insulin resistance
HR: hazard ratio
IA1c: impaired hemoglobin A1c
IFG: impaired fasting glucose
LDL-C: lowdensity lipoprotein cholesterol
MetS: Metabolic syndrome
ROS: reactive oxygen species
SBP: systolic blood pressure
TG: triglycerides

## References

[1] Busher J. Serum albumin and globulin In: Walker HK, Hall WD, Hurst JW, editors. Clinical Methods: The History, Physical, and Laboratory Examinations. Boston: Butterworths; 1990 pp. 497–499. 21250045

[2] Sitar ME, Aydin S, Cakatay U. Human serum albumin and its relation with oxidative stress. Clin Lab. 2013;59: 945–952 24273915

[3] Roche M, Rondeau P, Singh NR, Tarnus E, Bourdon E. The antioxidant properties of serum albumin. FEBS Lett. 2008;582: 1783–1787. 10.1016/j.febslet.2008.04.057 18474236

[4] Candiano G, Petretto A, Bruschi M, Santucci L, Dimuccio V, Prunotto M, et al The oxido-redox potential of albumin methodological approach and relevance to human diseases. J Proteomics. 2009;73: 188–195. 10.1016/j.jprot.2009.06.006 19540948

[5] Abbasi A, Bakker SJ, Corpeleijn E, van der A D, Gansevoort RT, Gans RO, et al Liver function tests and risk prediction of incident type 2 diabetes: evaluation in two independent cohorts. PLoS One. 2012;7: e51496 10.1371/journal.pone.0051496 23284703 PMC3524238

[6] Stranges S, Rafalson LB, Dmochowski J, Rejman K, Tracy RP, Trevisan M, et al Additional contribution of emerging risk factors to the prediction of the risk of type 2 diabetes: evidence from the Western New York Study. Obesity (Silver Spring). 2008;16: 1370–1376. 18356828 10.1038/oby.2008.59

[7] Schmidt MI, Duncan BB, Sharrett AR, Lindberg G, Savage PJ, Offenbacher S, et al Markers of inflammation and prediction of diabetes mellitus in adults (Atherosclerosis Risk in Communities study): a cohort study. Lancet. 1999;353: 1649–1652 10335783 10.1016/s0140-6736(99)01046-6

[8] Kunutsor SK, Khan H, Laukkanen JA. Serum albumin concentration and incident type 2 diabetes risk: new findings from a population-based cohort study. Diabetologia. 2015;58: 961–967. 10.1007/s00125-015-3520-0 25680582

[9] Cho HM, Kim HC, Lee JM, Oh SM, Choi DP, Suh I. The association between serum albumin levels and metabolic syndrome in a rural population of Korea. J Prev Med Public Health. 2012;45: 98–104. 10.3961/jpmph.2012.45.2.98 22509450 PMC3324721

[10] Ishizaka N, Ishizaka Y, Nagai R, Toda E, Hashimoto H, Yamakado M. Association between serum albumin, carotid atherosclerosis, and metabolic syndrome in Japanese individuals. Atherosclerosis. 2007;193: 373–379. 10.1016/j.atherosclerosis.2006.06.031 16904116

[11] Jin SM, Hong YJ, Jee JH, Bae JC, Hur KY, Lee MK, et al Change in serum albumin concentration is inversely and independently associated with risk of incident metabolic syndrome. Metabolism. 2016;65: 1629–1635. 10.1016/j.metabol.2016.08.006 27733251

[12] Ohn JH, Kwak SH, Cho YM, Lim S, Jang HC, Park KS, et al 10-year trajectory of beta-cell function and insulin sensitivity in the development of type 2 diabetes: a community-based prospective cohort study. Lancet Diabetes Endocrinol. 2016;4: 27–34. 10.1016/S2213-8587(15)00336-8 26577716

[13] Morimoto A, Tatsumi Y, Deura K, Mizuno S, Ohno Y, Miyamatsu N, et al Impact of impaired insulin secretion and insulin resistance on the incidence of type 2 diabetes mellitus in a Japanese population: the Saku study. Diabetologia. 2013;56: 1671–1679. 10.1007/s00125-013-2932-y 23680915

[14] Alberti KG, Eckel RH, Grundy SM, Zimmet PZ, Cleeman JI, Donato KA, et al Harmonizing the metabolic syndrome: a joint interim statement of the International Diabetes Federation Task Force on Epidemiology and Prevention; National Heart, Lung, and Blood Institute; American Heart Association; World Heart Federation; International Atherosclerosis Society; and International Association for the Study of Obesity. Circulation. 2009;120: 1640–1645. 10.1161/CIRCULATIONAHA.109.192644 19805654

[15] Bonora E, Kiechl S, Willeit J, Oberhollenzer F, Egger G, Targher G, et al Prevalence of insulin resistance in metabolic disorders: the Bruneck Study. Diabetes. 1998;47: 1643–1649 9753305 10.2337/diabetes.47.10.1643

[16] Matthews DR, Hosker JP, Rudenski AS, Naylor BA, Treacher DF, Turner RC. Homeostasis model assessment: insulin resistance and beta-cell function from fasting plasma glucose and insulin concentrations in man. Diabetologia. 1985;28: 412–419 3899825 10.1007/BF00280883

[17] Ford ES, Li C, Sattar N. Metabolic syndrome and incident diabetes: current state of the evidence. Diabetes Care. 2008;31: 1898–1904. 10.2337/dc08-0423 18591398 PMC2518368

[18] Halliwell B. Albumin—an important extracellular antioxidant? Biochem Pharmacol. 1988;37: 569–571 3277637 10.1016/0006-2952(88)90126-8

[19] Gutteridge JM. Antioxidant properties of the proteins caeruloplasmin, albumin and transferrin. A study of their activity in serum and synovial fluid from patients with rheumatoid arthritis. Biochim Biophys Acta. 1986;869: 119–127 3942755 10.1016/0167-4838(86)90286-4

[20] Galassetti P. Inflammation and oxidative stress in obesity, metabolic syndrome, and diabetes. Exp Diabetes Res. 2012;2012: 943706 10.1155/2012/943706 23319940 PMC3540748

[21] Dohi Y, Takase H, Sato K, Ueda R. Association among C-reactive protein, oxidative stress, and traditional risk factors in healthy Japanese subjects. Int J Cardiol. 2007;115: 63–66. 10.1016/j.ijcard.2006.04.006 16759717

[22] Park S, Kim M, Paik JK, Jang YJ, Lee SH, Lee JH. Oxidative stress is associated with C-reactive protein in nondiabetic postmenopausal women, independent of obesity and insulin resistance. Clin Endocrinol (Oxf). 2013;79: 65–70. 22816656 10.1111/j.1365-2265.2012.04512.x

[23] Abramson JL, Hooper WC, Jones DP, Ashfaq S, Rhodes SD, Weintraub WS, et al Association between novel oxidative stress markers and C-reactive protein among adults without clinical coronary heart disease. Atherosclerosis. 2005;178: 115–121. 10.1016/j.atherosclerosis.2004.08.007 15585208

[24] Maschirow L, Khalaf K, Al-Aubaidy HA, Jelinek HF. Inflammation, coagulation, endothelial dysfunction and oxidative stress in prediabetes—Biomarkers as a possible tool for early disease detection for rural screening. Clin Biochem. 2015;48: 581–585. 10.1016/j.clinbiochem.2015.02.015 25753569

[25] Nwose EU, Jelinek HF, Richards RS, Kerr PG. Changes in the erythrocyte glutathione concentration in the course of diabetes mellitus. Redox Rep. 2006;11: 99–104. 10.1179/135100006X116583 16805963

[26] Bossard M, Aeschbacher S, Schoen T, Hochgruber T, von Rotz M, Blum J, et al Serum bilirubin levels and risk of prediabetes in young and healthy adults. Int J Cardiol. 2014;171: e24–25. 10.1016/j.ijcard.2013.11.125 24360163

[27] Sudhindra RM, John SB. A study of serum uric acid in diabetes mellitus and prediabetes in a south Indian tertiary care hospital. Nitte Univ J Health Sci. 2012;2: 18–23

[28] Gillespie KM. Type 1 diabetes: pathogenesis and prevention. Cmaj. 2006;175: 165–170. 10.1503/cmaj.060244 16847277 PMC1489998

