# Supplementary material for: Increase in serum albumin concentration is associated with prediabetes development and progression to overt diabetes independently of metabolic syndrome
Source: PLoS One. 2017 Apr 21;12(4):e0176209. doi: 10.1371/journal.pone.0176209 (PMC5400249; doi:10.1371/journal.pone.0176209)
Supplement: S1 Fig — (DOCX) [file pone.0176209.s001.docx]

**S1 Fig. Selection of study subjects**

**Excluded (n = 13,393)**

- Baseline type 2 DM (n = 1321)and prediabetes (n = 4736)
- Baseline metabolic syndrome (n = 962)
- Diagnosis of type 2 DM (n = 28) or prediabetes

(n = 2053) within one year of follow-up

- Diagnosis of type 2 DM before diagnosis of prediabetes (n = 23)
- History of CVD (n = 692)
- Under age 20 (n = 4)
- Abnormal liver function
- TB/AST/ALT x 2 upper limit (n = 338)
- Positive HbsAg (n = 955)
- Positive HCV Ab (n = 191)
- Abnormal kidney function
- eGFR under 60 ml/min/1.73 m^2^ (n = 180)
- Use of lipid-lowering drug (n = 662)
- Missing data
- BMI (n = 5)
- SBP and DBP (n = 10)
- Lipid profile (n = 1)
- HbA1c (n = 21)
- Body and abdominal fat ratio (n = 39)
- Final albumin (n = 1211)

**Final participants without baseline prediabetes/DM and MetS**

**(n = 10,792)**

24,185 subjects were assessed for eligibility

Normal glucose tolerance

(n = 5,409)

Incident prediabetes

(n = 4,398)

**`**

**cro**

Incident MetS during follow-up (n = 985)
