# Supplementary material for: Increase in serum albumin concentration is associated with prediabetes development and progression to overt diabetes independently of metabolic syndrome
Source: PLoS One. 2017 Apr 21;12(4):e0176209. doi: 10.1371/journal.pone.0176209 (PMC5400249; doi:10.1371/journal.pone.0176209)
Supplement: S1 Table — (DOCX) [file pone.0176209.s002.docx]

**S1 Table. Clinical characteristics according to baseline serum albumin levels**

| Variables | Quartile 1 | Quartile 2 | Quartile 3 | Quartile 4 | p value |
| --- | --- | --- | --- | --- | --- |
|  | < 4.1 mg/dl | 4.1 - 4.2 mg/dl | 4.3 – 4.4 mg/dl | > 4.4 mg/dl |  |
| Number of subjects | 1557 | 2825 | 3190 | 2235 |  |
| Age (year) | 50.46 ± 7.89 | 49.72 ± 7.75 | 49.02 ± 7.61 | 47.49 ± 7.59 | < 0.001 |
| Male (n, %) | 504 (32.4) | 1148 (40.6) | 1659 (52.0) | 1317 (58.9) | < 0.001 |
| Waist circumference (cm) | 77.87 ± 8.68 | 78.55 ± 8.45 | 79.88 ± 8.67 | 80.33 ± 8.64 | < 0.001 |
| BMI (kg/m^2^) | 22.70 ± 2.35 | 22.58 ± 2.38 | 22.77 ± 2.54 | 22.70 ± 2.51 | 0.035 |
| Body fat (%) | 24.80 ± 6.16 | 24.21 ± 6.17 | 23.61 ± 6.13 | 23.21 ± 5.54 | < 0.001 |
| Hypertension (n, %) | 114 (7.3) | 202 (7.2) | 229 (7.2) | 178 (8.0) | 0.418 |
| Current smoker (n, %) | 170 (10.9) | 352 (12.5) | 467 (14.6) | 329 (14.7) | < 0.001 |
| Fasting glucose (mg/dl) | 82.96 ± 6.80 | 84.01 ± 6.73 | 84.79 ± 6.88 | 85.43 ± 6.87 | < 0.001 |
| HbA1c (%) | 5.16 ± 0.26 | 5.15 ± 0.27 | 5.16 ± 0.26 | 5.15 ± 0.27 | 0.374 |
| HOMA-IR (n = 6,222) | 1.63 ± 0.68 | 1.67 ± 0.68 | 1.74 ± 0.69 | 1.78 ± 0.74 | < 0.001 |
| ALT (U/l) | 17.02 ± 7.99 | 18.03 ± 8.49 | 20.01 ± 9.75 | 21.57 ± 10.89 | < 0.001 |
| Total cholesterol (mg/dl) | 178.44 ± 29.75 | 185.12 ± 29.81 | 189.22 ± 30.12 | 194.65 ± 31.26 | < 0.001 |
| LDL-C (mg/dl) | 111.68 ± 26.30 | 117.00 ± 26.54 | 120.90 ± 27.02 | 125.23 ± 28.20 | < 0.001 |
| HDL-C (mg/dl) | 60.83 ± 13.60 | 61.49 ± 13.85 | 60.80 ± 13.74 | 61.34 ± 13.93 | 0.167 |
| TG (mg/dl) | 93.88 ± 43.92 | 96.57 ± 46.77 | 106.21 ± 57.54 | 109.25 ± 53.92 | < 0.001 |
| eGFR (ml/min/1.73m^2^) | 89.21 ± 12.70 | 89.55 ± 12.06 | 90.39 ± 12.13 | 90.50 ± 12.35 | 0.001 |
| CRP (mg/dl) (n = 9,789) | 0.05 (0.03-0.10) | 0.05 (0.03-0.09) | 0.05 (0.03-0.09) | 0.05 (0.03-0.08) | 0.005 |
| Incidence of prediabetes, n (%) | 678 (43.5) | 1225 (43.4) | 1522 (47.7) | 973 (43.5) | 0.283 |

Data are presented as mean ± SD, median (25^th^ to 75^th^ percentile), or percentage.

p value was calculated from one way analysis of variance (ANOVA) or the Kruskal-Wallis test for continuous variables or the Chi-square test for categorical variables.

Abbreviations: BMI, body mass index; HOMA-IR, homeostatic model assessment-insulin resistance; ALT, alanine aminotransferase; LDL-C, low density lipoprotein cholesterol, HDL-C;high density lipoprotein cholesterol; TG, triglyceride; eGFR, estimated glomerular filtration rate; CRP, C-reactive protein.
