# Supplementary material for: Increase in serum albumin concentration is associated with prediabetes development and progression to overt diabetes independently of metabolic syndrome
Source: PLoS One. 2017 Apr 21;12(4):e0176209. doi: 10.1371/journal.pone.0176209 (PMC5400249; doi:10.1371/journal.pone.0176209)
Supplement: S2 Table — (DOCX) [file pone.0176209.s003.docx]

**S2 Table.** **Hazard ratios of serum albumin change for prediabetes development according to age subgroup**

|  | **Baseline age ( < 60 years)** | | **Baseline age ( ≥ 60 years)** | |
| --- | --- | --- | --- | --- |
|  | Hazard ratio (95% CI) | p value | Hazard ratio (95% CI) | p value |
| Crude | 0.933 (0.929-0.938) | < 0.001 | 0.932 (0.918-0.946) | < 0.001 |
| Model 1 | 0.936 (0.931-0.940) | < 0.001 | 0.932 (0.918-0.946) | < 0.001 |
| Model 2 | 0.942 (0.937-0.947) | < 0.001 | 0.945 (0.931-0.959) | < 0.001 |
| Model 3 | 0.942 (0.937-0.947) | < 0.001 | 0.945 (0.931-0.959) | < 0.001 |
| Model 4 | 0.942 (0.937-0.947) | < 0.001 | 0.945 (0.931-0.960) | < 0.001 |
| Model 5 | 0.939 (0.933-0.945) | < 0.001 | 0.942 (0.923-0.963) | < 0.001 |

Data are expressed as hazard ratio (95% confidence interval).

Model 1: adjusted for gender, and BMI

Model 2: adjusted for Model 1 plus fasting plasma glucose and HbA1c

Model 3: adjusted for Model 2 plus ALT, TG, LDL-C, HDL-C, eGFR, hypertension and smoking status.

Model 4: adjusted for Model 3 plus logCRP (n = 9.789).

Model 5: adjusted for Model 4 plus HOMA-IR (n = 6,222).
