# Supplementary material for: Increase in serum albumin concentration is associated with prediabetes development and progression to overt diabetes independently of metabolic syndrome
Source: PLoS One. 2017 Apr 21;12(4):e0176209. doi: 10.1371/journal.pone.0176209 (PMC5400249; doi:10.1371/journal.pone.0176209)
Supplement: S3 Table — (DOCX) [file pone.0176209.s004.docx]

**S3 Table. Hazard ratios and 95% confidence intervals for prediabetes development according to percent change in serum albumin level after adjusting for both BMI and BMI change**

| Serum albumin  percent change | Quartile 1  (< - 2.08%) | Quartile 2  (-2.08 – 2.43 %) | Quartile 3  (2.44 – 7.31 %) | Quartile 4  ( > 7.31 %) | p for trend | Cont.variable  (per 1%) | p value |
| --- | --- | --- | --- | --- | --- | --- | --- |
| Crude | 1 | 0.61 (0.57-0.66) | 0.43 (0.40-0.47) | 0.34 (0.31-0.37) | < 0.001 | 0.933 (0.928-0.937) | < 0.001 |
| Multivariate 1 | 1 | 0.63 (0.58-0.68) | 0.44 (0.41-0.48) | 0.36 (0.33-0.40) | < 0.001 | 0.936 (0.932-0.941) | < 0.001 |
| Multivariate 2 | 1 | 0.68 (0.63-0.73) | 0.49 (0.45-0.53) | 0.42 (0.38-0.45) | < 0.001 | 0.944 (0.939-0.948) | < 0.001 |
| Multivariate 3 | 1 | 0.68 (0.63-0.73) | 0.49 (0.45-0.53) | 0.42 (0.38-0.45) | < 0.001 | 0.944 (0.939-0.948) | < 0.001 |
| Multivariate 4 | 1 | 0.68 (0.63-0.73) | 0.49 (0.45-0.53) | 0.41 (0.38-0.45) | < 0.001 | 0.943 (0.939-0.948) | < 0.001 |
| Multivariate 5 | 1 | 0.68 (0.61-0.74) | 0.47 (0.42-0.52) | 0.39 (0.35-0.43) | < 0.001 | 0.941 (0.935-0.946) | < 0.001 |

Data are expressed as hazard ratio (95% confidence interval).

Model 1: adjusted for age, gender, BMI and BMI change

Model 2: adjusted for Model 1 plus fasting plasma glucose and HbA1c

Model 3: adjusted for Model 2 plus ALT, TG, LDL-C, HDL-C, eGFR, smoking status, and hypertension.

Model 4: adjusted for Model 3 plus log CRP (n = 9,789).

Model 5: adjusted for Model 4 plus HOMA-IR (n = 6,222).
