# Supplementary material for: Increase in serum albumin concentration is associated with prediabetes development and progression to overt diabetes independently of metabolic syndrome
Source: PLoS One. 2017 Apr 21;12(4):e0176209. doi: 10.1371/journal.pone.0176209 (PMC5400249; doi:10.1371/journal.pone.0176209)
Supplement: S4 Table — (DOCX) [file pone.0176209.s005.docx]

**S4 Table. Comparison between the effect of change in serum albumin and conventional metabolic risk factors on the risk for prediabetes**

| Covariates | Univariate model | | Multivariate model^a^ | | | |
| --- | --- | --- | --- | --- | --- | --- |
|  | HR (95% CI) | p value | | │Z│^b^ | HR (95% CI) | p value |
| Age | 1.04 (1.03-1.04) | < 0.001 | | 6.00 | 1.02 (1.01-1.03) | < 0.001 |
| Sex (male) | 1.54 (1.45-1.63) | < 0.001 | | 0.26 | 1.01 (0.91-1.13) | 0.790 |
| BMI | 1.12 (1.10-1.13) | < 0.001 | | 5.89 | 1.06 (1.04-1.07) | < 0.001 |
| Fasting glucose | 1.06 (1.05-1.06) | < 0.001 | | 10.00 | 1.03 (1.02-1.04) | < 0.001 |
| HbA1c | 13.50 (11.81-15.44) | < 0.001 | | 23.5 | 7.74 (6.52-9.18) | < 0.001 |
| ALT | 1.02 (1.01-1.02) | < 0.001 | | 0.50 | 1.00 (0.99-1.01) | 0.512 |
| TG | 1.003 (1.002-1.004) | < 0.001 | | 0.30 | 1.001 (1.000-1.001) | 0.001 |
| LDL | 1.007 (1.006-1.008) | < 0.001 | | 0.50 | 1.000 (0.998-1.001) | 0.781 |
| HDL | 0.991 (0.989-0.994) | < 0.001 | | 0.50 | 0.999 (0.996-1.002) | 0.477 |
| eGFR | 0.99 (0.98-0.99) | < 0.001 | | 0.50 | 1.00 (0.98-1.00) | 0.725 |
| Hypertension | 1.59 (1.44-1.76) | < 0.001 | | 2.00 | 1.15 (1.01-1.31) | 0.045 |
| Smoking (current) | 1.46 (1.34-1.58) | < 0.001 | | 2.97 | 1.21 (1.07-1.36) | 0.003 |
| HOMA-IR | 1.24 (1.18-1.30) | < 0.001 | | 0.38 | 1.01 (0.96-1.07) | 0.704 |
| Log CRP | 1.30 (1.21-1.40) | < 0.001 | | 0.18 | 1.01 (0.92-1.11) | 0.851 |
| % change (per 1%) of albumin | 0.93 (0.92-0.94) | < 0.001 | | 20.67 | 0.94 (0.93-0.95) | < 0.001 |

^a^Multivariate model was adjusted for age, sex, BMI, fasting glucose, HbA1c, ALT, TG, LDL, HDL, eGFR, hypertension, smoking,

HOMA-IR, and log CRP.

^b^Z is the coefficient divided by its standard error. Larger values indicate greater significance.
